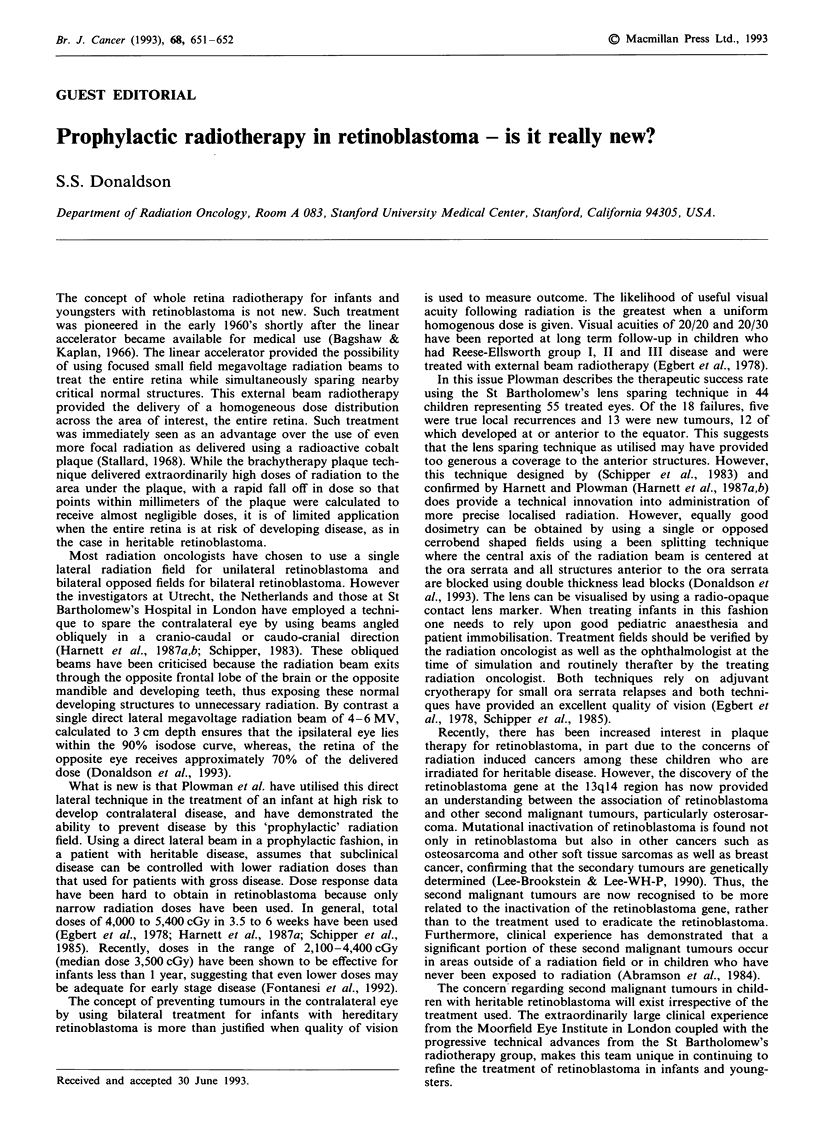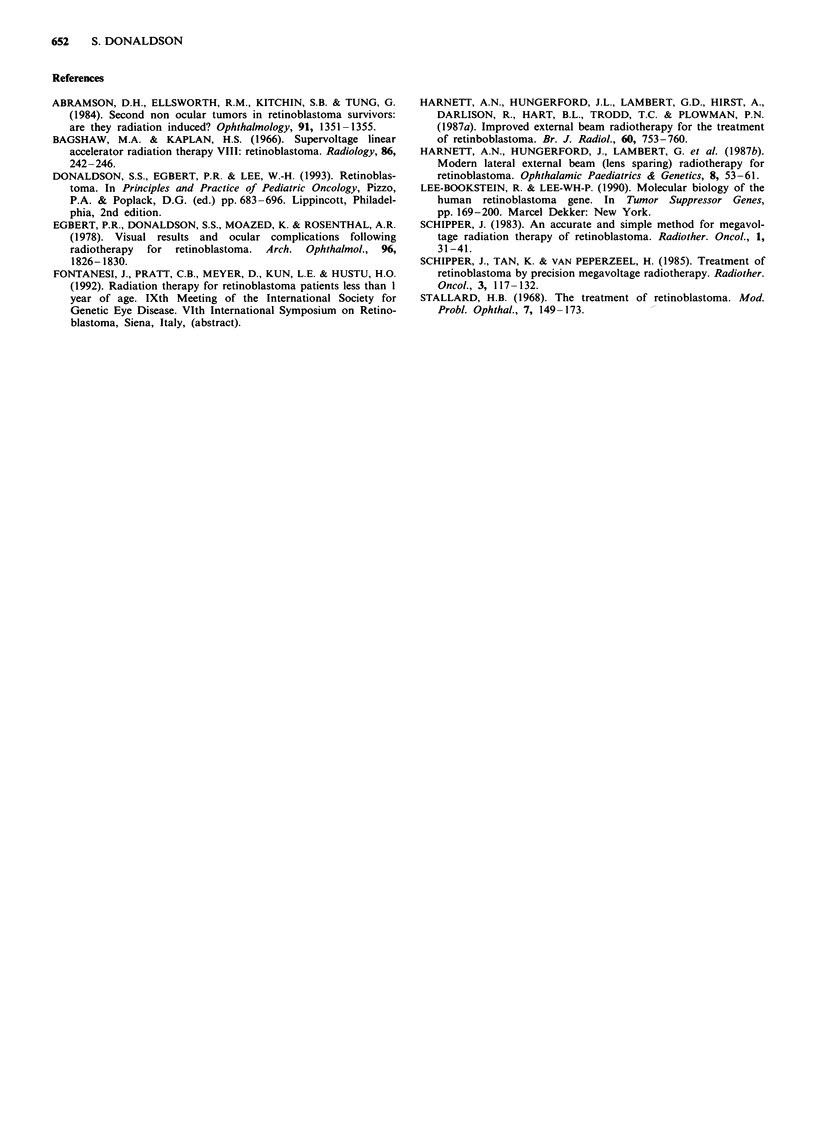# Prophylactic radiotherapy in retinoblastoma--is it really new?

**DOI:** 10.1038/bjc.1993.403

**Published:** 1993-10

**Authors:** S. S. Donaldson


Br. J. Cancer (1993), **68**, 651–652 © Macmillan Press Ltd., 1993

GUEST EDITORIAL

### Prophylactic radiotherapy in retinoblastoma – is it really new?

S.S. Donaldson

*Department of Radiation Oncology, Room A 083, Stanford University Medical Center, Stanford, California 94305, USA.*

The concept of whole retina radiotherapy for infants and youngsters with retinoblastoma is not new. Such treatment was pioneered in the early 1960's shortly after the linear accelerator became available for medical use (Bagshaw & Kaplan, 1966). The linear accelerator provided the possibility of using focused small field megavoltage radiation beams to treat the entire retina while simultaneously sparing nearby critical normal structures. This external beam radiotherapy provided the delivery of a homogeneous dose distribution across the area of interest, the entire retina. Such treatment was immediately seen as an advantage over the use of even more focal radiation as delivered using a radioactive cobalt plaque (Stallard, 1968). While the brachytherapy plaque technique delivered extraordinarily high doses of radiation to the area under the plaque, with a rapid fall off in dose so that points within millimeters of the plaque were calculated to receive almost negligible doses, it is of limited application when the entire retina is at risk of developing disease, as in the case in heritable retinoblastoma.

Most radiation oncologists have chosen to use a single lateral radiation field for unilateral retinoblastoma and bilateral opposed fields for bilateral retinoblastoma. However the investigators at Utrecht, the Netherlands and those at St Bartholomew's Hospital in London have employed a technique to spare the contralateral eye by using beams angled obliquely in a cranio-caudal or caudo-cranial direction (Harnett *et al.*, 1987*a,b*; Schipper, 1983). These obliqued beams have been criticised because the radiation beam exits through the opposite frontal lobe of the brain or the opposite mandible and developing teeth, thus exposing these normal developing structures to unnecessary radiation. By contrast a single direct lateral megavoltage radiation beam of 4–6 MV, calculated to 3 cm depth ensures that the ipsilateral eye lies within the 90% isodose curve, whereas, the retina of the opposite eye receives approximately 70% of the delivered dose (Donaldson *et al.*, 1993).

What is new is that Plowman *et al.* have utilised this direct lateral technique in the treatment of an infant at high risk to develop contralateral disease, and have demonstrated the ability to prevent disease by this 'prophylactic' radiation field. Using a direct lateral beam in a prophylactic fashion, in a patient with heritable disease, assumes that subclinical disease can be controlled with lower radiation doses than that used for patients with gross disease. Dose response data have been hard to obtain in retinoblastoma because only narrow radiation doses have been used. In general, total doses of 4,000 to 5,400 cGy in 3.5 to 6 weeks have been used (Egbert *et al.*, 1978; Harnett *et al.*, 1987*a*; Schipper *et al.*, 1985). Recently, doses in the range of 2,100–4,400 cGy (median dose 3,500 cGy) have been shown to be effective for infants less than 1 year, suggesting that even lower doses may be adequate for early stage disease (Fontanesi *et al.*, 1992).

The concept of preventing tumours in the contralateral eye by using bilateral treatment for infants with hereditary retinoblastoma is more than justified when quality of vision is used to measure outcome. The likelihood of useful visual acuity following radiation is the greatest when a uniform homogenous dose is given. Visual acuities of 20/20 and 20/30 have been reported at long term follow-up in children who had Reese-Ellsworth group I, II and III disease and were treated with external beam radiotherapy (Egbert *et al.*, 1978).

In this issue Plowman describes the therapeutic success rate using the St Bartholomew's lens sparing technique in 44 children representing 55 treated eyes. Of the 18 failures, five were true local recurrences and 13 were new tumours, 12 of which developed at or anterior to the equator. This suggests that the lens sparing technique as utilised may have provided too generous a coverage to the anterior structures. However, this technique designed by (Schipper *et al.*, 1983) and confirmed by Harnett and Plowman (Harnett *et al.*, 1987*a,b*) does provide a technical innovation into administration of more precise localised radiation. However, equally good dosimetry can be obtained by using a single or opposed cerrobend shaped fields using a been splitting technique where the central axis of the radiation beam is centered at the ora serrata and all structures anterior to the ora serrata are blocked using double thickness lead blocks (Donaldson *et al.*, 1993). The lens can be visualised by using a radio-opaque contact lens marker. When treating infants in this fashion one needs to rely upon good pediatric anaesthesia and patient immobilisation. Treatment fields should be verified by the radiation oncologist as well as the ophthalmologist at the time of simulation and routinely therafter by the treating radiation oncologist. Both techniques rely on adjuvant cryotherapy for small ora serrata relapses and both techniques have provided an excellent quality of vision (Egbert *et al.*, 1978, Schipper *et al.*, 1985).

Recently, there has been increased interest in plaque therapy for retinoblastoma, in part due to the concerns of radiation induced cancers among these children who are irradiated for heritable disease. However, the discovery of the retinoblastoma gene at the 13q14 region has now provided an understanding between the association of retinoblastoma and other second malignant tumours, particularly osterosarcoma. Mutational inactivation of retinoblastoma is found not only in retinoblastoma but also in other cancers such as osteosarcoma and other soft tissue sarcomas as well as breast cancer, confirming that the secondary tumours are genetically determined (Lee-Brookstein & Lee-WH-P, 1990). Thus, the second malignant tumours are now recognised to be more related to the inactivation of the retinoblastoma gene, rather than to the treatment used to eradicate the retinoblastoma. Furthermore, clinical experience has demonstrated that a significant portion of these second malignant tumours occur in areas outside of a radiation field or in children who have never been exposed to radiation (Abramson *et al.*, 1984).

The concern regarding second malignant tumours in children with heritable retinoblastoma will exist irrespective of the treatment used. The extraordinarily large clinical experience from the Moorfield Eye Institute in London coupled with the progressive technical advances from the St Bartholomew's radiotherapy group, makes this team unique in continuing to refine the treatment of retinoblastoma in infants and youngsters.

Received and accepted 30 June 1993.

### References

ABRAMSON, D.H., ELLSWORTH, R.M., KITCHIN, S.B. & TUNG, G. (1984). Second non ocular tumors in retinoblastoma survivors: are they radiation induced? *Ophthalmology*, **91**, 1351–1355.

BAGSHAW, M.A. & KAPLAN, H.S. (1966). Supervoltage linear accelerator radiation therapy VIII: retinoblastoma. *Radiology*, **86**, 242–246.

DONALDSON, S.S., EGBERT, P.R. & LEE, W.-H. (1993). Retinoblastoma. In *Principles and Practice of Pediatric Oncology*, Pizzo, P.A. & Poplack, D.G. (ed.) pp. 683–696. Lippincott, Philadelphia, 2nd edition.

EGBERT, P.R., DONALDSON, S.S., MOAZED, K. & ROSENTHAL, A.R. (1978). Visual results and ocular complications following radiotherapy for retinoblastoma. *Arch. Ophthalmol.*, **96**, 1826–1830.

FONTANESI, J., PRATT, C.B., MEYER, D., KUN, L.E. & HUSTU, H.O. (1992). Radiation therapy for retinoblastoma patients less than 1 year of age. IXth Meeting of the International Society for Genetic Eye Disease. VIth International Symposium on Retinoblastoma, Siena, Italy, (abstract).

HARNETT, A.N., HUNGERFORD, J.L., LAMBERT, G.D., HIRST, A., DARLISON, R., HART, B.L., TRODD, T.C. & PLOWMAN, P.N. (1987*a*). Improved external beam radiotherapy for the treatment of retinboblastoma. *Br. J. Radiol.*, **60**, 753–760.

HARNETT, A.N., HUNGERFORD, J., LAMBERT, G. *et al.* (1987*b*). Modern lateral external beam (lens sparing) radiotherapy for retinoblastoma. *Ophthalamic Paediatrics & Genetics*, **8**, 53–61.

LEE-BOOKSTEIN, R. & LEE-WH-P. (1990). Molecular biology of the human retinoblastoma gene. In *Tumor Suppressor Genes*, pp. 169–200. Marcel Dekker: New York.

SCHIPPER, J. (1983). An accurate and simple method for megavoltage radiation therapy of retinoblastoma. *Radiother. Oncol.*, **1**, 31–41.

SCHIPPER, J., TAN, K. & VAN PEPERZEEL, H. (1985). Treatment of retinoblastoma by precision megavoltage radiotherapy. *Radiother. Oncol.*, **3**, 117–132.

STALLARD, H.B. (1968). The treatment of retinoblastoma. *Mod. Probl. Ophthal.*, **7**, 149–173.